# Anti-inflammatory Role of Carotenoids in Endothelial Cells Derived from Umbilical Cord of Women Affected by Gestational Diabetes Mellitus

**DOI:** 10.1155/2019/8184656

**Published:** 2019-01-30

**Authors:** Mariangela Ucci, Pamela Di Tomo, Federica Tritschler, Vincenzo G. P. Cordone, Paola Lanuti, Giuseppina Bologna, Sara Di Silvestre, Natalia Di Pietro, Caterina Pipino, Domitilla Mandatori, Gloria Formoso, Assunta Pandolfi

**Affiliations:** ^1^Department of Medical, Oral and Biotechnological Sciences, “G. d'Annunzio” University Chieti-Pescara, Chieti, Italy; ^2^Centro Scienze dell'Invecchiamento-Medicina Traslazionale, CeSI-MeT, “G. d'Annunzio” University Chieti-Pescara, Chieti, Italy; ^3^Department of Medicine and Aging Sciences, “G. d'Annunzio” University Chieti-Pescara, Chieti, Italy

## Abstract

Diabetes is associated with vascular inflammation, endothelial dysfunction, and oxidative stress, promoting the development of cardiovascular diseases (CVD). Several studies showed that a carotenoid-rich diet is associated to a reduced cardiovascular risk in healthy and diabetic subjects, although the mechanisms of action are still unknown. Here, the potential role of *β*-carotene (BC) and lycopene (Lyc) in human endothelial cells isolated from human umbilical cord vein (HUVECs) of women with gestational diabetes (GD) and respective controls (C) has been investigated. Results showed that BC and Lyc reduced the tumor necrosis factor alpha- (TNF-*α*-) stimulated monocyte-endothelium interaction (adhesion assay), membrane exposure (flow cytometry), and total expression levels (Western blot) of VCAM-1 and ICAM-1 in both cell types. Moreover, the treatment with BC and Lyc reduced the TNF-*α*-induced nuclear translocation of NF-*κ*B (image flow cytometry) by preserving bioavailability of nitric oxide (NO, flow cytometry, and cGMP EIA kit assay), a key vasoactive molecule. Notably, BC and Lyc pretreatment significantly reduced peroxynitrite levels (flow cytometry), contributing to the redox balance protection. These results suggest a new mechanism of action of carotenoids which exert vascular protective action in diabetic condition, thus reinforcing the importance of a carotenoid-rich diet in the prevention of diabetes cardiovascular complications.

## 1. Introduction

Cardiovascular diseases represent the major complications and the main cause of reduced life expectancy in type 2 diabetic patients [1].

Diabetes is a chronic low-grade inflammatory condition featured by the increased plasma levels of TNF-*α*, a primary mediator of inflammation and insulin resistance [2], and reactive oxygen species (ROS), both playing an important role in the promotion of endothelial dysfunction and cardiovascular complications [3].

Nitric oxide is an important molecule playing a pleiotropic role in preserving vascular wall homeostasis. It is produced by endothelial nitric oxide synthase (eNOS) via the conversion of the amino acid L-arginine into L-citrulline. Once released, it diffuses to the vascular smooth muscle cells (vSMC), where it activates the enzyme guanylate cyclase (GC), inducing the production of cyclic guanosine monophosphate (cGMP), a molecule involved in vascular relaxation. Moreover, nitric oxide (NO) modulates platelet aggregation and monocyte adhesion and infiltration into the vascular wall and inhibits vSMC proliferation and migration [4, 5]. Thus, the maintenance of nitric oxide availability is mandatory to avoid the activation of the inflammatory process and the endothelial dysfunction.

However, several studies found that under hyperglycemic conditions NO rapidly reacts with superoxide anion (O_2_^•−^) to form peroxynitrite (ONOO^−^), a highly potent oxidant molecule that diffuses across phospholipid membranes, resulting in substrate nitrosylation and nitric oxide bioavailability decline [6–8].

Several natural molecules seem to have a beneficial effect on oxidative stress and vascular dysfunction [9–11]; among them, carotenoids are the most characterized. Carotenoids include a large family of fat-soluble molecules noted for their antioxidant action [12]. However, besides their antioxidant effect, they also exert an anti-inflammatory effect, playing an important role in the prevention of cardiovascular complications [13, 14].

Among more than 700 carotenoids discovered, the main and better characterized are *β*-carotene and lycopene with free radical scavenger activity and nutritional relevance [15]. Interestingly, several studies showed that carotenoids are able not only to prevent but also to ameliorate diabetes and its subsequent complications by reducing oxidative stress [16, 17].

In preclinical reports using streptozotocin- (STZ-) induced hyperglycemic rats as a model to evaluate the effect of chronic lycopene treatment [18–20], it was found that this molecule acts as an antidiabetic agent, attenuating endothelial dysfunction by its antioxidant action.

Moreover, other studies also indicated that dietary lycopene administration markedly reduced serum lipid levels and the formation of atherosclerotic plaques in New Zealand White (NZW) rabbits fed a high-fat diet [21–23], further indicating that lycopene could play a significant role in the prevention of cardiovascular consequences.

As regard human studies, although the evidence on the beneficial effects of carotenoids in the reduction of diabetes incidence is controversial [24–26], it was demonstrated that serum concentration of carotenoids was inversely associated with future oxidative stress, inflammation, and endothelial dysfunction [27]. In addition, prospective investigations highlighted that adequate dietary intakes of carotenoids were associated with a reduced risk for type 2 diabetes mellitus (T2DM) [28–30], inducing to establish a “carotenoid health index” to better evaluate the cardiovascular risk according to established plasmatic carotenoid concentrations [31].

Although several *in vitro* cellular models have been used to evaluate the role of carotenoids in reducing the development and the progression of the atherosclerotic plaque [17, 32, 33], it is however mandatory to better delineate the molecular events involved in the beneficial effect of carotenoids in slowing down the endothelial dysfunction and the atherosclerotic process in diabetes mellitus.

In this study, we used an endothelial cell model of chronic hyperglycemia derived from the human umbilical cord vein of women affected by gestational diabetes (GD-HUVECs). Recently, we found that these cells exhibit durable proatherogenic modifications of cellular homeostasis potentially predisposing to endothelial dysfunction and atherosclerosis development [34, 35], making them a useful model for studying endothelial dysfunction related to diabetes. Thus, we aim to investigate the molecular mechanisms of new natural molecules such as *β*-carotene and lycopene on the prevention or the delay of the vascular damage induced by hyperglycemia.

In particular, to better outline the way of action of carotenoids in endothelial dysfunction prevention in diabetes, we pretreated TNF-*α*-stimulated GD-HUVECs with *β*-carotene and lycopene. Interestingly, we found that the exposure of diabetic HUVECs to *β*-carotene and lycopene resulted in an increased nitric oxide bioavailability, probably induced by the scavenging action of carotenoids, and in the reduction of the oxidative and inflammatory stress damage.

## 2. Materials and Methods

### 2.1. Materials

Phosphate-buffered saline (PBS, CAT. D8662), Dulbecco's modified Eagle medium (DMEM, CAT. D6046), M199 endothelial growth medium (CAT. M4530), 0.5% trypsin/0.2%, ethylenediaminetetraacetic acid (EDTA) solution (CAT. 59418C), bovine serum albumin (BSA), L-glutamine (CAT. G7513), penicillin-streptomycin (CAT. P4333), phorbol myristate acetate (PMA, CAT. P1585), ionomycin (Iono, CAT. I0634), anti-*β*-actin mouse monoclonal antibody (CAT. A5441), 7-aminoactinomycin D (7-AAD, CAT. A9400), and TNF-*α* (CAT. T0157) were purchased from Sigma-Aldrich (Saint Louis, USA). Fetal bovine serum (FBS, CAT. 41A0045K) was from Life Technologies (Monza, Italy), and L-nitro-arginine-methyl ester (L-NAME, CAT. ALX-105-003) was purchased from Alexis Biochemicals (San Diego, CA, USA). Anti-vascular cell adhesion molecule-1 (VCAM-1, CAT. sc-13160) and anti-intercellular adhesion molecule-1 (ICAM-1, CAT. sc-107) antibodies were from Santa Cruz Biotechnology (Santa Cruz, CA, USA). PE-labeled anti-VCAM-1 (phycoerythrin-labeled, CAT. 305806) and FITC-labeled anti-ICAM-1 (fluorescein isothiocyanate-labeled, CAT. 313104) antibodies were from BioLegend (San Diego, CA, USA). Anti-NF-*κ*B p65 (CAT. 4764) primary antibody was from Cell Signaling (Danvers, MA, USA). Alexa Fluor 488-conjugated antibody was from Invitrogen (CAT. 11034). DAF-2DA probe was from Calbiochem (CAT. 251505). HKGreen-4A was synthesized by Prof. Dan Yang's lab [36]. An enzyme immunoassay (EIA) kit was taken from GE Healthcare (Little Chalfont, Buckinghamshire, UK). Lycopene (Lyc, CAT. L9879) was purchased from Sigma-Aldrich, and *β*-carotene (BC, CAT. 22040) from Fluka (Hamburg, Germany): both were dissolved in tetrahydrofuran (THF, CAT. 401757, Sigma-Aldrich) and used as described in our previous work [37].

### 2.2. Cell Cultures and Experimental Protocol

Primary human umbilical vein endothelial cells (HUVECs) were explanted by umbilical cords obtained from randomly selected mothers affected by gestational diabetes (GD) and healthy Caucasian mothers (Control, C), according to the previously reported methods [38]. The characteristics of C-mothers (*n* = 10) and GD-mothers (*n* = 12) selected for this work are described in Table 1. All procedures were in agreement with the ethical standards of the Institutional Committee on Human Experimentation (reference number 1879/09COET) and with the Declaration of Helsinki principles. For experiments, C- and GD-HUVECs were grown to subconfluence in a DMEM/M199 medium (ratio 1 : 1) supplemented with 20% FBS, 10 *μ*g/mL heparin and 50 *μ*g/mL endothelial cell growth factor. Serum-starved cells (in medium with 0.5% FBS) were incubated with TNF-*α* at concentration 1 ng/mL for 16 hours, following 24-hour preincubation with *β*-carotene or lycopene (2.5 *μ*mol/L).

All experiments were performed in technical duplicate or triplicate using at least 3 different cellular strains (*n* = 3) obtained from umbilical cords of C- or GD-women.

### 2.3. Monocyte-HUVEC Adhesion Assays

The adhesion assay was performed in C- and GD-HUVECs in the basal state and after incubation for 24 hours with BC or Lyc (2.5 *μ*mol/L) before stimulation with or without 1 ng/mL TNF-*α* for 16 hours. The cells were grown to confluence in six-well tissue culture plates and U937 cell lines (European Collection of Authenticated Cell Cultures (ECACC)) were used to evaluate the adhesion to HUVEC monolayers as previously described [39]. One hour before the assay, HUVECs were treated with antibodies against VCAM-1 or ICAM-1 at saturating concentrations (1 *μ*g/1 × 10^6^ cells) as negative technical controls. Photos were randomly chosen high-power fields taken at a half-radius distance from the centre of the well in one of three comparative experiments of a similar design, showing U937 monocytoid cell adhesion to endothelial cells.

### 2.4. Western Blot Analysis

C- and GD-HUVECs were stimulated as described in the experimental protocol, and Western blot analysis was performed as previously described [10]. For the specific experiment, cells were lysed and 30 *μ*g total protein was resolved by SDS-PAGE, transferred to nitrocellulose membrane, and immunoblotted using mouse monoclonal anti-VCAM-1 and ICAM-1 (1 : 1000 and 1 : 500, respectively) and mouse monoclonal anti-*β*-actin (1 : 10.000). The membranes were then incubated with peroxidase-conjugated secondary antibodies (1 : 10.000). Band densities of proteins were detected and quantified by using the Alliance Chemiluminescence Imaging System (UVItec Limited, Cambridge, United Kingdom). Densities of VCAM-1 and ICAM-1 proteins were divided by those of *β*-actin content, and the ratio was indicated as arbitrary units.

### 2.5. cGMP EIA Kit Assay

Control and GD-HUVECs were grown to confluence in six-well tissue culture plates and were stimulated as described in the experimental protocol. To stimulate endogenous NO production, C- and GD-HUVECs were incubated with ionomycin (2 *μ*mol/L for 24 h) with or without L-NAME preincubation (1 mmol/L for 45 minutes). Intracellular cGMP levels were evaluated by using a commercial enzyme immunoassay (EIA) kit, following the instruction provided by the supplier.

### 2.6. Flow Cytometry Analysis

At the basal state and after stimulations, nonpermeabilized cells were detached by 5 mM EDTA, washed, and resuspended in 0.5% BSA solution. Cells were treated and incubated with anti-VCAM-1 PE conjugate (1 : 100) and with anti-ICAM-1 FITC conjugate (1 : 100) as previously described [38]. To determine cytoplasm-nucleus translocation of NF-*κ*B p65, cells were permeabilized by an Intrasure kit (CAT. 641778, BD Biosciences), processed, and incubated with anti-NF-*κ*B p65 (1 : 100) primary antibody and then secondary antibody Alexa 488 conjugate (1 : 100). Nuclear staining was performed by incubating the cells with 7-AAD (1 : 100) for 10 minutes at room temperature, and all samples were analysed by imaging flow cytometry (ImageStream AMNIS by using IDEAS software, BD).

To evaluate NO levels, 5 × 10^5^ C- and GD-HUVECs were incubated with the cell-permeable fluorescent nitric oxide probe DAF-2DA (2 *μ*mol/L for 30 minutes at 37°C). For the evaluation of intracellular levels of peroxynitrite (ONOO^−^), about 5 × 10^5^ cells were incubated with the HKGreen-4A probe as previously described [38]. 10.000 events for each sample were analysed using a FACS Calibur or FACS Canto II flow cytometer (BD Bioscences, California, USA). All data were analysed using FACS Diva (BD Bioscences), FlowJo v.8.8.6 (TreeStar, Ashland, OR), and CELL Quest 3.2.1 software (BD Biosciences).

All results are expressed as the percentage (%) of positive cells or the MFI (mean fluorescence intensity) ratio, calculated by dividing the MFI of positive events by the MFI of negative events (MFI of secondary antibody).

### 2.7. Statistical Analysis

Results are presented as the means ± standard deviation (SD) of at least 3 different experiments using at least 3 different cellular strains (*n* = 3) both of C-HUVECs and of GD-HUVECs. Student's *t*-test and ANOVA test followed by the Bonferroni multiple comparison test for post hoc comparisons were used to analyse the differences between the two cell strains and between the different treatments. Significance was defined as a *p* value less than 0.05.

## 3. Results

### 3.1. Effect of Carotenoids on Monocyte-HUVEC Interaction

The effects of carotenoids on human monocyte line U937 adhesion rate to control and GD-HUVECs, in basal or TNF-*α*-stimulated conditions, were investigated.

**Figure 1:**
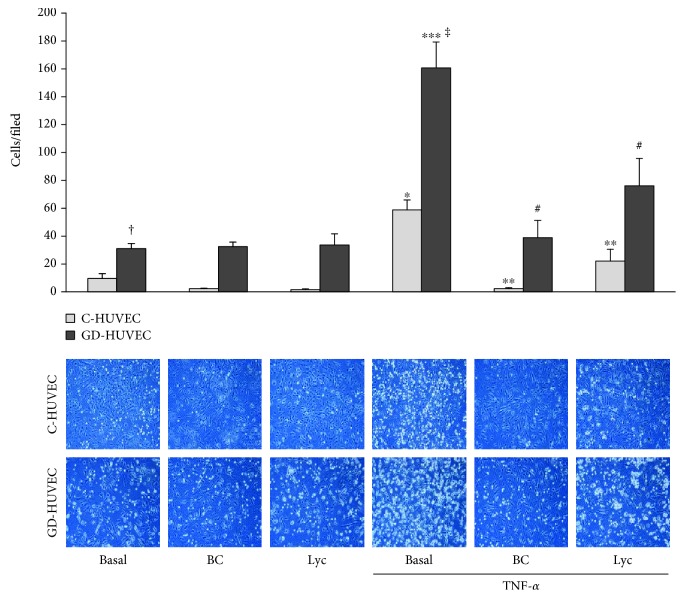
Effect of carotenoids on TNF-*α*-induced monocyte interaction in C- and GD-HUVECs. Monocyte-HUVEC adhesion in C- and GD-HUVECs untreated (Basal) and incubated for 24 h with BC or Lyc (2.5 *μ*mol/L) and then stimulated for 16 h with or without TNF-*α* (1 ng/mL). In the histogram (upper side), quantitative data express the number of U937 cells adhering within a high-power field (3.5mm^2^). Each measurement is expressed as the mean ± SD of adhering cells from 3 experiments (*n* = 3), each consisting of 8 counts per condition. In the lower side, representative photos of C- and GD-HUVECs for each experimental condition. *ANOVA and Bonferroni multiple comparison test:*^∗^*p* < 0.05 vs. basal C-HUVECs, ^∗∗^*p* < 0.05 vs. TNF-*α* C-HUVECs, ^∗∗∗^*p* < 0.05 vs. Basal GD-HUVECs, ^#^*p* < 0.05 vs. TNF-*α* GD-HUVECs. *Student'st-test:*^†^*p* < 0.0002 basal GD-HUVECs vs. basal C-HUVECs, ^‡^*p* < 0.0001 TNF-*α* GD-HUVECs vs. TNF-*α* C-HUVECs.

Figure 1 shows that, in the basal state, the monocyte-GD-HUVEC interaction was significantly higher compared to C-HUVECs (*p* < 0.0002). The exposure to 1 ng/mL TNF-*α* further increased this difference (*p* < 0.05). Interestingly, pretreatment with 2.5 *μ*mol/L of BC or Lyc for 24 hours significantly resulted in the reduction of monocyte adhesion induced by TNF-*α* to both cell types (*p* < 0.05).

### 3.2. Effect of Carotenoids on Adhesion Molecule Membrane Exposure and Expression

The exposure of the adhesion molecules on the endothelial cell membrane is the major mechanism responsible for the monocyte-endothelial cell interaction. We thus evaluated VCAM-1 and ICAM-1 membrane exposure and total protein expression in C- and GD-HUVECs with or without the pretreatment with BC or Lyc (2.5 *μ*mol/L for 24 h) and in the presence or absence of the inflammatory stimulus TNF-*α*.

**Figure 2:**
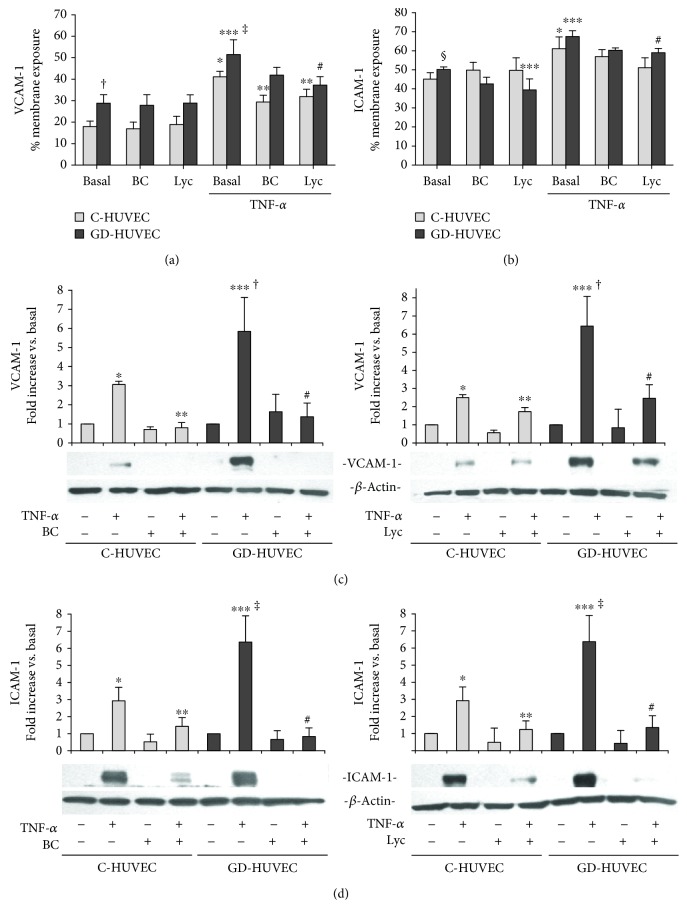
The effect of carotenoids on adhesion molecule membrane exposure and total expression after TNF-*α*-stimulation in C- and GD-HUVECs. VCAM-1 (a) and ICAM-1 (b) membrane exposure in C- and GD-HUVECs untreated (basal) and incubated for 24 h with BC or Lyc (2.5 *μ*mol/L) and then stimulated for 16 h with or without TNF-*α* (1 ng/mL). Quantitative data in histograms result from 4 different experiments (*n* = 4). The results are expressed as the percentage of positive cells for surface exposure on the plasma membrane of VCAM-1 and ICAM-1 in not permeabilized cells. Representative Western blot and its histogram for VCAM-1 (c) and ICAM-1 (d) total protein expression in C- and GD-HUVECs untreated (Basal) and incubated for 24 h with 2.5 *μ*mol/L of BC (left panels) or Lyc (right panels) and then stimulated for 16 h with or without TNF-*α* (1 ng/mL). Quantitative data in histograms result from 3 different experiments (*n* = 3). The results for the VCAM-1 or ICAM-1 and *β*-actin ratio are expressed as arbitrary units, and data are shown as fold increase vs. basal condition of the mean ± SD from three independent experiments. *ANOVA and Bonferroni multiple comparison test:*^∗^*p* < 0.05 vs. basal C-HUVECs, ^∗∗^*p* < 0.05 vs. TNF-*α* C-HUVECs, ^∗∗∗^*p* < 0.05 vs. basal GD-HUVECs, ^#^*p* < 0.05 vs. TNF-*α* GD-HUVECs. *Student'st-test:* in (a) and (b), ^†^*p* < 0.001 and ^§^*p* = 0.05 basal GD-HUVECs vs. basal C-HUVECs, ^‡^*p* < 0.05 TNF-*α* GD-HUVECs vs. TNF-*α* C-HUVECs; in (c) and (d), ^†^*p* < 0.03 and ^‡^*p* < 0.05 TNF-*α* GD-HUVECs vs. TNF-*α* C-HUVECs.

Figure 2 shows that both basal VCAM-1 (Figure 2(a)) and ICAM-1 (Figure 2(b)) exposure is greater on GD-HUVEC membrane compared to control cells (*p* < 0.001 and *p* = 0.05, respectively). TNF-*α* increased the exposure of VCAM-1 and ICAM-1 in both cell types (*p* < 0.05). The increased exposure induced by TNF-*α* was significantly reduced in the presence of 2.5 *μ*mol/L for 24 h BC or Lyc (*p* < 0.05). Interestingly, in GD-HUVECs, Lyc is able to reduce ICAM-1 exposure on the endothelial membrane also in the basal state (*p* < 0.05).

After 16 h of 1 ng/mL TNF-*α* stimulation, a significant increase in VCAM-1 (Figure 2(c)) and ICAM-1 (Figure 2(d)) total protein levels was observed. The increase was more pronounced in GD-HUVECs as compared to C-HUVECs (*p* < 0.05). Remarkably, in TNF*α*-stimulated C- and GD-HUVECs, the pretreatment with 2.5 *μ*mol/L for 24 h of BC (left) or Lyc (right) significantly decreased adhesion molecule protein levels (*p* < 0.05), supporting the idea of the potential role played by these carotenoids in the reduction of the monocyte adhesion.

### 3.3. Effect of Carotenoids on NF-*κ*B p65 Nuclear Translocation

**Figure 3:**
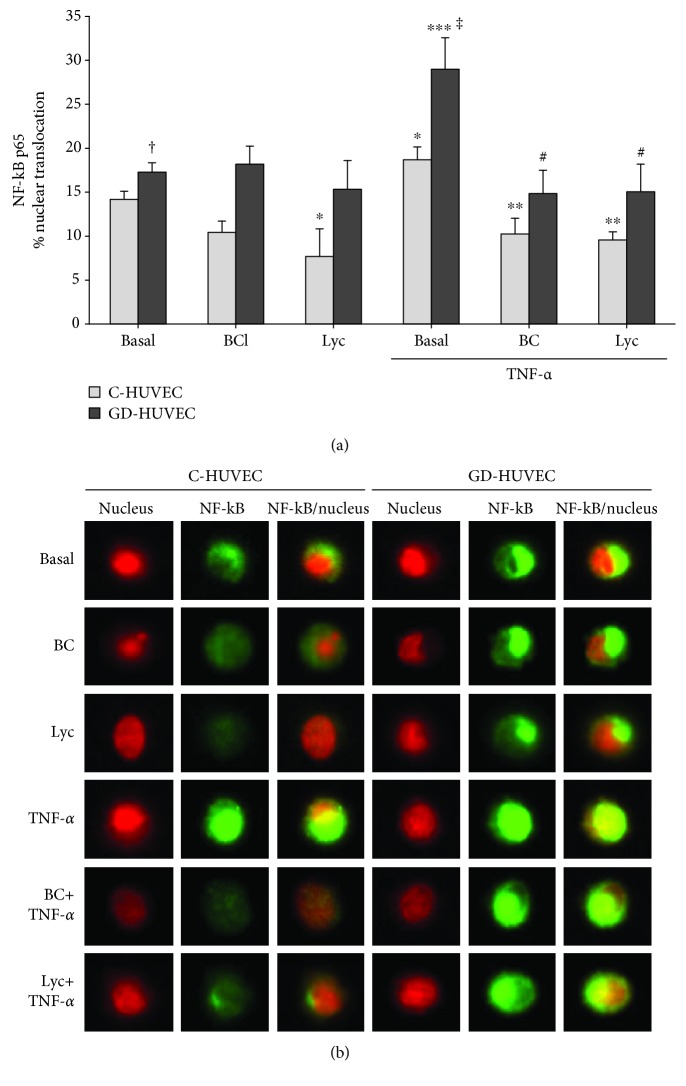
The effect of carotenoids on TNF-*α*-increased NF-*κ*B p65 nuclear translocation levels in C- and GD-HUVECs. The histogram (a) and representative single-cell images (b) of NF-*κ*B p65 cytoplasm-nucleus translocation in untreated (basal) or TNF-*α* stimulated C- and GD-HUVECs after preincubation for 24 h with of BC or Lyc (2.5 *μ*mol/L). In (a), data in the histogram result from 3 independent experiments (*n* = 3) and are expressed as the percentage of positive cells for nucleus-NF-*κ*B p65 colocalization. In (b), nuclei are stained in red and NF-*κ*B p65 in green for each experimental condition. *ANOVA and Bonferroni multiple comparison test:*^∗^*p* < 0.05 vs. basal C-HUVECs, ^∗∗^*p* < 0.05 vs. TNF-*α* C-HUVECs, ^∗∗∗^*p* < 0.05 vs. basal GD-HUVECs, ^#^*p* < 0.05 vs. TNF-*α* GD-HUVECs. *Student'st-test*: ^†^*p* < 0.01 basal GD-HUVECs vs. basal C-HUVECs, ^‡^*p* < 0.005 TNF-*α* GD-HUVECs vs. TNF-*α* C-HUVECs.

Figure 3 shows the nucleus-NF-*κ*B p65 colocalization rate expressed as the histogram (Figure 3(a)) and single-cell images (Figure 3(b)) in the presence or absence of BC and Lyc (2.5 *μ*mol/L for 24 h) in C- and GD-HUVECs with or without the inflammatory stimulus TNF-*α*.

As expected, compared to C-HUVECs, GD-HUVECs at basal condition showed an enhanced NF-*κ*B p65 nuclear translocation level (*p* < 0.01), which was further significantly increased following TNF-*α* stimulation in both cell types (*p* < 0.05), and it resulted to be more evident in GD-HUVECs (*p* < 0.005 vs. C-HUVECs). Interestingly, 24 h pretreatment with BC or Lyc was associated to a significant reduction of TNF-*α*-induced NF-*κ*B nuclear translocation in both GD- and C-HUVECs (*p* < 0.05). It is noteworthy how in BC and Lyc pre-treated control cells a reduction of NF-*κ*B nuclear translocation was also evident in basal condition.

### 3.4. Effect of Carotenoids on NO Bioavailability

**Figure 4:**
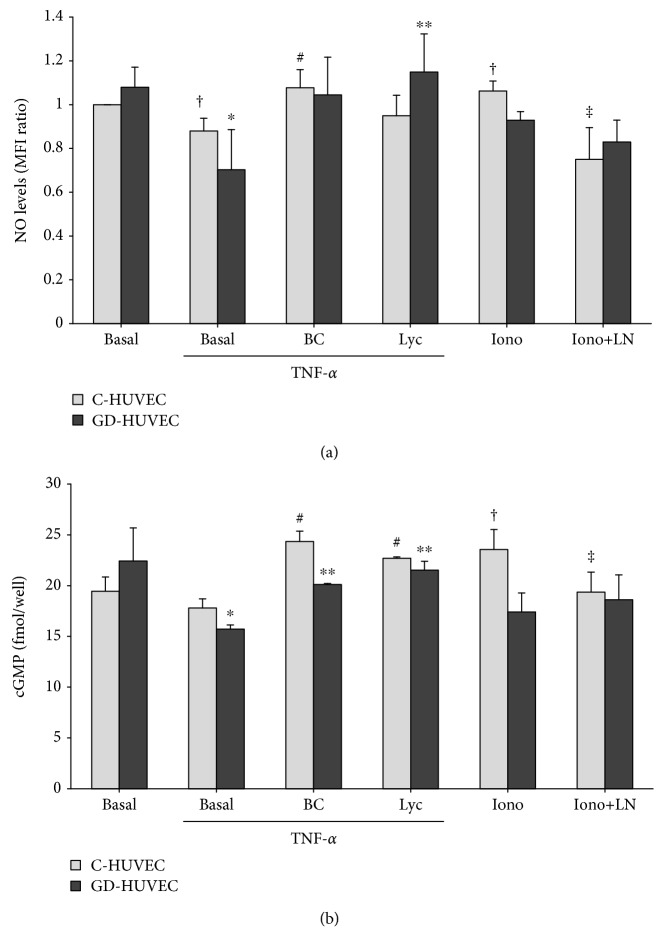
The effects of carotenoids on NO bioavailability in C- and GD-HUVECs. (a) Nitric oxide generation measured by DAF-2DA cytometric analysis and (b) cGMP levels measured by an EIA kit in HUVECs pretreated with BC or Lyc (2.5 mmol/L) in the presence or absence of 16 h stimulation with TNF-*α* (1 ng/mL). The stimulation with ionomycin (Iono, 2 *μ*mol/L) for 24 h with or without L-NAME (LN, 1 mmol/L) preincubation (45 minutes) is used as a positive control. In (a), data are expressed as the mean fluorescence intensity (MFI) ratio (signal to noise ratio) from 4 independent experiments (*n* = 4). In (b), data result from 3 different experiments (*n* = 3) and are expressed as fmol/well. *ANOVA and Bonferroni multiple comparison test:*^∗^*p* < 0.05 vs. basal and ^∗∗^*p* < 0.05 vs. TNF-*α* in GD-HUVECs, ^#^*p* < 0.05 vs. TNF-*α* C-HUVECs. *Student'st-test*: ^†^*p* < 0.05 vs. basal and ^‡^*p* < 0.05 vs. Iono C-HUVECs.

As shown in Figure 4, as compared to basal condition, TNF-*α* stimulation significantly decreased nitric oxide levels both in C- and in GD-HUVECs (*p* < 0.05). Pretreatment with 2.5 *μ*mol/L *β*-carotene and lycopene restored NO bioavailability in both the cell types. Moreover, after TNF-*α* exposure, both the cell types displayed decreased levels of cGMP, a biological target of NO activity, compared to their basal condition, and this was more evident in GD-HUVECs (*p* < 0.05). Notably, cGMP content significantly increased after pretreatment with BC and Lyc in both TNF-*α*-stimulated C-HUVECs and TNF-*α*-stimulated GD-HUVECs (*p* < 0.05). As a positive control, eNOS activator ionomycin (Iono) stimulation was used and a significant increase in nitric oxide bioavailability and cGMP levels (*p* < 0.05) was observed in C-HUVECs, the effect that was abolished by the preincubation with the eNOS inhibitor L-NAME (*p* < 0.05).

### 3.5. Effect of Carotenoids on Peroxynitrite Production

To better understand if the increased NO levels after BC and Lyc stimulation were associated with a reduced peroxynitrite formation, we evaluated the intracellular ONOO^−^ production in C- and GD-HUVECs in the presence or absence of an inflammatory stimulus.

**Figure 5:**
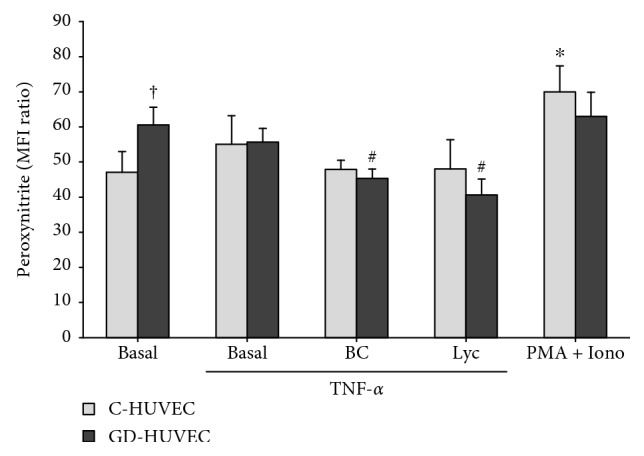
The effect of carotenoids on peroxynitrite levels in C- and GD-HUVECs. Intracellular peroxynitrite production in C- and GD-HUVECs incubated for 24 h with BC or Lyc (2.5 *μ*mol/L) with or without TNF-*α*-stimulation (1 ng/mL) for 16 h. Data in the histogram are expressed as the mean fluorescence intensity (MFI) ratio (signal to noise ratio) of 4 independent experiments (*n* = 4). Phorbol myristate acetate (PMA, 200 ng/mL) and ionomycin (Iono, 50 nM) for 30 min before the assay are used as positive controls for endogenous peroxynitrite production. *ANOVA and Bonferroni multiple comparison test:*^∗^*p* < 0.05 vs. basal C-HUVECs, ^#^*p* < 0.05 vs. TNF-*α* GD-HUVECs. *Student'st-test*: ^†^*p* < 0.05 basal GD-HUVECs vs. basal C-HUVECs.

Figure 5 shows that, in the basal state, GD-HUVECs display greater peroxynitrite levels compared to C-HUVECs (*p* < 0.05). The pretreatment with 2.5 *μ*mol/L of BC or Lyc for 24 h decreased the peroxynitrite levels in both TNF*α*-stimulated cell types. This result is more evident in GD-HUVECs (*p* < 0.05). The potent oxidant molecule PMA (200 ng/mL) and Ionomycin (50 nmol/L) pretreatment for 30 minutes highly increased the percentage of positive cells for peroxynitrite probe (*p* < 0.05), mostly in control with respect to GD-HUVECs, confirming the efficiency of the assay.

## 4. Discussion

Cardiovascular complications are the major consequences of chronic hyperglycemia and represent the main reason for impaired life expectancy in diabetic patients [40]. In fact, cardiovascular events represent the most prevalent cause of morbidity and mortality in diabetic patients [40]; thus, an optimal control of both hyperglycemia and cardiovascular risk factors is necessary to prevent adverse outcomes in type 2 diabetic patients [41]. More in depth knowledge of the complex of mechanisms controlling vascular damage in diabetes suggested that some natural molecules could be able to address multiple aspects of diabetes and its complications and, most importantly, to reduce disease-related morbidity and mortality [14].

Although several studies have been performed regarding the potential protective role of some natural antioxidant molecules, disparate results have been obtained regarding the beneficial effect of antioxidant therapy in the reduction of diabetes incidence and the prevention of its cardiovascular complications [42].

Carotenoids are among the main characterized natural antioxidants studied in order to find new potential protective molecules for chronic inflammation and oxidative stress [12]. However, controversial data have been found on their effects. Indeed, it is likely that the structure of carotenoids makes these molecules highly susceptible to oxidation under certain conditions, such as the oxygen partial pressure (PO_2_) and their high amount [43–46], inducing a reduction in their nutritional value and their beneficial action [47, 48].

In addition, several studies also showed that the mechanisms of action and the antioxidant capacities of carotenoids could be totally different and strongly dependent upon their interaction with other antioxidant compounds [49]. Indeed, an increase in the concentration of one might reduce the absorption of another with a great antioxidant capacity reducing the overall effectiveness [50]. In fact, the effect of antioxidant molecules has to be considered the result of a complex network involving several reactive species and other biological targets, and the redox regulation has to consider not only the ROS imbalance in a quantitative manner but also their chemical structure, cellular location, formation and degradation rate, and physiological functions [51]. Furthermore, the presence of chemical interactions between reactive oxygen species, which influence the response of organism to environmental challenges and stressors ensuring its homeostasis, must be considered. Then, it is necessary not to discard the idea that inappropriate removal of ROS could be also be self-defeating [52].

Hence, there is still not a definite scenery regarding the potential benefic effect of carotenoid diet administration in diabetes cardiovascular complication prevention and their mechanism of action, so further analyses are needed.

In the present study, we investigated the mechanisms potentially involved in carotenoid prevention of vascular inflammation and atherogenesis under chronic hyperglycemic condition.

In particular, we evaluated the effect of *β*-carotene and lycopene on the modulation of the inflammatory and nitro-oxidative state of GD-HUVECs, which represent a useful cellular model of endothelial dysfunction occurring during hyperglycemic conditions [34]. Cells were exposed *in vitro* to *β*-carotene and lycopene concentration comparable to circulating levels of those molecules reached after oral administration, *in vivo*.

The results obtained suggest a new hypothesis regarding the mechanism of action of carotenoids in the prevention of diabetes-related cardiovascular complications.

At first, in order to evaluate carotenoids' anti-inflammatory action, we determined the monocyte-endothelial cell interaction rate, finding that *β*-carotene and lycopene significantly reduced monocyte-HUVEC adhesion in TNF-*α*-stimulated C- and GD-HUVECs (Figure 1). Coherently, we also observed that BC and Lyc significantly decreased TNF-*α*-induced VCAM-1 and ICAM-1 membrane exposure and the total protein expression in both control and GD cells (Figure 2).

Moreover, it is noted that NF-*κ*B plays a fundamental role in the expression of proinflammatory molecules such as cytokines, chemokine, and adhesion molecules [53] and several studies demonstrated that it is highly involved in metabolic disorders and atherosclerosis [54, 55], thus evaluating the effect of carotenoids treatment on NF-*κ*B nuclear translocation.

Interestingly, we found that, under a TNF-*α*-stimulated state, carotenoid treatment significantly reduced NF-*κ*B nuclear translocation in control cells as well as in GD-endothelial cells (Figure 3). Notably, these data highlight the ability of carotenoids to inhibit the inflammatory pathway not only in healthy conditions, as previously found [37], but also in a hyperglycemic state, suggesting their anti-inflammatory role in diabetes.

In this regard, we consider the effect of BC and Lyc pretreatment in the modulation of nitric oxide bioavailability, which is involved in the modulation of the NF-*κ*B pathway [56] and thus in the vascular homeostasis balance.

Of note, in endothelial cells chronically exposed to high glucose and inflammation, despite an increase in NO production [57], the bioavailability of nitric oxide is decreased, as we previously demonstrated [34], probably as results of the “quenching” of NO, which rapidly reacts with the high levels of superoxide to produce peroxynitrite [58].

Here, we further confirm that the exposition to the proinflammatory stimulus TNF-*α* promotes a decline in NO levels and a decrease of the production of cGMP, its biological target, both in C- and in GD-HUVECs (Figure 4). However, remarkably, we also found that the exposure to *β*-carotene and lycopene induced an increase in NO bioavailability, particularly in GD-HUVECs.

In this regard, not surprisingly, the pretreatment with BC and Lyc resulted in the decreased peroxynitrite production in both TNF-*α*-exposed control and GD-HUVECs (Figure 5), confirming their antioxidant action and their role in the promotion of nitric oxide level maintenance.

All together, these results indicate that carotenoids contribute to restore endothelial homeostasis in an endothelial cell model chronically exposed to high-glucose levels by promoting nitric oxide bioavailability, exerting both antioxidant and anti-inflammatory actions.

## 5. Conclusion

In conclusion, data obtained in the present study elucidate the mechanisms of action of carotenoids in the modulation of the inflammatory and oxidative state induced *in vitro* by the proinflammatory molecule TNF-*α* on an endothelial cellular model of chronic hyperglycemia.

Then, while care must be taken regarding the safety of chronic and high-dose carotenoid supplementation, our results show that a diet amount administration of these natural food components could be important for the management of the vascular homeostasis in hyperglycemic conditions, speculating that a carotenoid-rich diet could prevent cardiovascular complications in diabetic patients.

## Figures and Tables

**Table 1:** Clinical characteristics of control (C, *n* = 10) and gestational diabetic (GD, *n* = 12) women.

Characteristic	C-women	GD-women
Age (years)	35 ± 7.1	34 ± 5.67
Height (cm)	163.75 ± 5.66	162.4 ± 7.93
Pregestational weight (kg)	68.14 ± 13	67.1 ± 10.73
BMI (kg/m^2^)	27.49 ± 5.18	27.81 ± 2.97
*OGTT values* (*mmol/L*)		
Basal glycaemia	4.5 ± 0.24	5.1 ± 0.24^∗∗^
1 h glycaemia	8.1 ± 0.99	10.2 ± 1.16^∗∗^
2 h glycaemia	6.54 ± 1.14	8.04 ± 1.71^∗^
OGTT gestational week	27.9 ± 2.4	24.4 ± 4.7
SBP (mm/Hg)	107.6 ± 8.87	105.5 ± 10.7
DBP (mm/Hg)	71.4 ± 9.1	68.4 ± 10.57

Data are expressed as the mean ± SD. BMI: body mass index, OGTT: oral glucose tolerance test, SBP: systolic blood pressure, DBP: diastolic blood pressure. ^∗∗^*p* < 0.05; ^∗^*p* < 0.0001.

## Data Availability

The experimental data and materials used to support the findings of this study are available from the corresponding author upon request.
